# UClncR: Ultrafast and comprehensive long non-coding RNA detection from RNA-seq

**DOI:** 10.1038/s41598-017-14595-3

**Published:** 2017-10-27

**Authors:** Zhifu Sun, Asha Nair, Xianfeng Chen, Naresh Prodduturi, Junwen Wang, Jean-Pierre Kocher

**Affiliations:** 1 Division of Biomedical Statistics and Informatics, Department of Health Sciences Research, Mayo Clinic, Rochester, MN 55905 USA; 2 Department of Health Sciences Research & Center for Individualized Medicine, Mayo Clinic, Scottsdale, AZ 85259 USA; 3 Department of Biomedical Informatics, Arizona State University, Scottsdale, AZ 85259 USA

## Abstract

Long non-coding RNA (lncRNA) is a large class of gene transcripts with regulatory functions discovered in recent years. Many more are expected to be revealed with accumulation of RNA-seq data from diverse types of normal and diseased tissues. However, discovering novel lncRNAs and accurately quantifying known lncRNAs is not trivial from massive RNA-seq data. Herein we describe UClncR, an Ultrafast and Comprehensive lncRNA detection pipeline to tackle the challenge. UClncR takes standard RNA-seq alignment file, performs transcript assembly, predicts lncRNA candidates, quantifies and annotates both known and novel lncRNA candidates, and generates a convenient report for downstream analysis. The pipeline accommodates both un-stranded and stranded RNA-seq so that lncRNAs overlapping with other genes can be predicted and quantified. UClncR is fully parallelized in a cluster environment yet allows users to run samples sequentially without a cluster. The pipeline can process a typical RNA-seq sample in a matter of minutes and complete hundreds of samples in a matter of hours. Analysis of predicted lncRNAs from two test datasets demonstrated UClncR’s accuracy and their relevance to sample clinical phenotypes. UClncR would facilitate researchers’ novel lncRNA discovery significantly and is publically available at http://bioinformaticstools.mayo.edu/research/UClncR.

## Introduction

Long non-coding RNAs (lncRNAs) are a new and large class of gene transcripts that do not code proteins. The number of known lncRNAs has significantly increased in recent years due to the broader adoption of high throughput sequencing platforms^1–3^. LncRNAs account for roughly 68% of transcribed genes^3,4^. The unique features of lncRNAs, for example, short lengths, regulatory functions and tissue specific expression, make them potential better biomarker candidates for disease diagnosis and prognosis^5,6^. As most of the newly discovered lncRNAs are from a limited number of tissue or cell types, it is expected that many new lncRNAs are yet to be characterized, particularly in diverse and heterogeneous human diseased tissues such as different types of human cancer.

New lncRNA candidates (or loosely “novel” lncRNAs) are generally discovered from RNA-sequencing (RNA-seq) data. The sequencing data has to undergo multiple processing steps, starting from sequence read alignment, transcript assembly, non-coding potential evaluation to noise filtering and expression quantification. There have been no streamlined and easy-to-use public pipelines for the process, particularly for a large number of samples. Sebnif (self-estimation based novel lincRNA filter pipeline) provides a certain degree of solution^7^. It takes pre-assembled transcripts (such as from Cufflinks) and predicts “novel” long intergenic non-coding RNAs (lincRNAs) for a sample. The major feature of this tool is the filtering of less reliable lincRNA candidates by their expression level using statistical models. Additionally, single exon lincRNA candidates are filtered out if they overlap too much with a repeat region of a reference genome. The non-coding potential evaluation is achieved by iSeeRNA^8^.

While Sebnif is a useful tool for one or few samples, it becomes inconvenient for a larger study with many samples. The tool needs pre-assembled transcript candidates, which is the most time consuming and computationally intensive operation. Cufflinks^9^ or Scripture^10^ is commonly used but they are very slow and not scalable for a large number of samples. Sebnif can only process one sample at time, which is not only cumbersome for a large study but also difficult to compare predicted lincRNAs across multiple samples as each sample has a different set of candidates. An integrated pipeline that combines all steps and works efficiently is needed.

To overcome these limitations and provide a convenient tool, we have developed an Ultrafast and Comprehensive lncRNA detection and quantification pipeline named UClncR by taking advantage of a super-fast transcript assembly tool, Sebnif’s features, and parallel computing. Firstly, UClncR incorporates the most recent and super-fast StringTie^11^ for transcript assembly as an integral part of the pipeline, which was demonstrated with better performances than its predecessors^11^. Secondly, in addition to iSeeRNA^8^ for noncoding potential evaluation, we also  added the internally developed and widely used CPAT^12^ for combined use of coding potential assessment (intersection or union) for increased sensitivity or specificity. Thirdly, UClncR predicts lncRNA candidates but keeps known (i.e., annotated) lncRNAs intact so that they can all be analyzed together. This is important as years of research have characterized many well-known lncRNAs and pure digital prediction may break known lncRNA structure and the link between studies, making across-comparison difficult. Fourthly, UClncR can process both stranded and non-stranded RNA-seq data. For non-stranded RNA-seq, it is only possible to predict and quantify lincRNAs; however, for stranded RNA-seq all lncRNAs (including overlapping transcripts) are characterized. Fifthly, when UClncR processes multiple samples together, it merges all predicted candidates and generates a unified lncRNA annotation for next step expression quantification. This allows easier comparison across samples such as differential expression between conditions. Lastly, UClncR can distribute jobs on a cluster of computers to process multiple samples simultaneously. Tens or hundreds of samples can be completed in a matter of hours, which dramatically facilitates the analysis for a large project.

## Methods

### Pipeline overview

**Figure 1:**
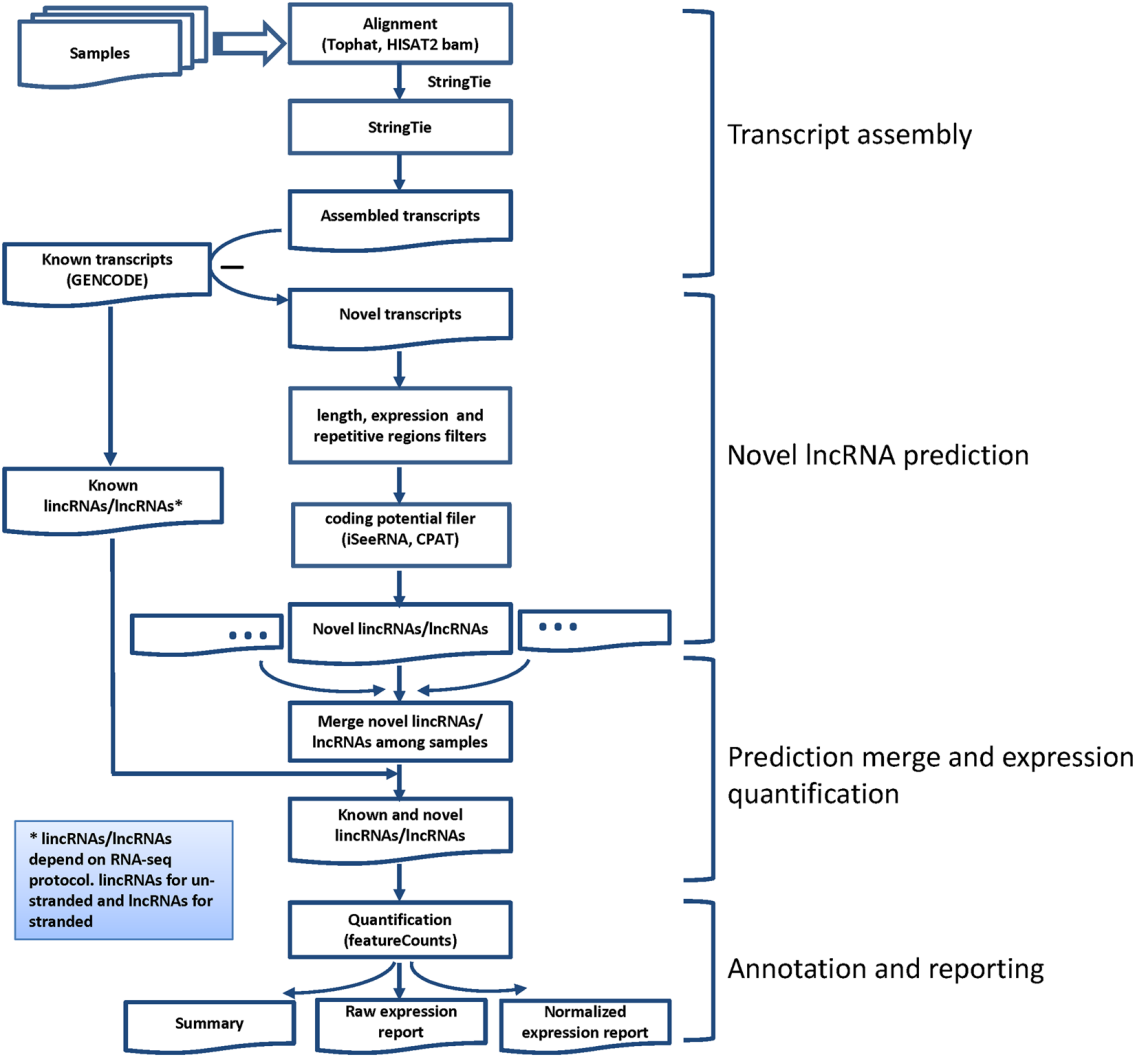
UClncR workflow diagram. The workflow starts from aligned bam (right parameters for stranded/unstranded RNA-seq should be set) for transcript assembly by StringTie. For un-stranded RNA-seq, the workflow only works with lincRNAs. Known lincRNAs are simply quantified and novel lincRNAs are predicted and quantified. For stranded RNA-seq, overlap transcripts in the opposite strand are quantified and predicted.

The pipeline has four major modules: de novo transcript assembly, lncRNA candidate prediction, known and lncRNA candidate quantification, and reporting (Fig. 1). The de novo transcript assembly starts from aligned RNA-seq so that transcript candidates can be built. The aligned bam needs to be produced by splice-junction aware aligners such as Tophat, Tophat2, HISAT2, or STAR (HISAT2 is preferred as it is faster than Tophat and more compatible with StringTie) with correct parameters for stranded RNA-seq. StringTie^11^ is used to perform transcript assembly. Once the transcript candidates are built, the pipeline predicts potential lncRNA through two different routes depending on the RNA-seq protocol of non-stranded or stranded as they are handled very differently. The third step of the pipeline is to quantify both known and predicted lncRNAs and the final step is to generate consolidated reports across samples with annotations. For non-strand specific RNA-seq, only lincRNAs are predicted and quantified; however for stranded RNA-seq, lncRNAs overlapping other transcripts in opposite strands are also predicted and quantified.

### Non-stranded RNA-seq

Non-strand specific RNA-seq is commonly used for short read sequencing; however, the coding strand information of a transcript is not kept in this data. As many lncRNAs are in the coding region of other genes but on the opposite strand, it is impossible to distinguish the expression of these lncRNAs from their overlapping genes. Therefore, only lncRNAs in the intergenic regions (or lincRNAs) can be predicted or quantified correctly in this data because they don’t have any overlap with other transcripts. UClncR performs following tasks for non-stranded RNA-seq data (Fig. 1): (1) Conducts non-strand specific transcript assembly by StringTie. (2) Predicts lincRNA candidates for each individual sample. (3) Merges predicted lincRNAs from multiple samples by the Cuffmerge function of the Cufflinks so that a common predicted lincRNA annotation file (GTF) is generated. This step is very important since a study frequently includes multiple samples and the unified assembly facilitates downstream comparison between samples. Additionally, when a project contains multiple samples, sequence depths can differ where samples with higher depths are more likely to have low expressed lncRNAs predicted. Using the consolidated annotation, we can quantify the expression of lncRNAs in the samples with lower sequence depths. More importantly, by comparing multiple assemblies from multiple samples, a consensus transcriptome with support from more samples is more reliable. Although Cuffmerge is used by default, users can select either the “merge” function of Stringtie or a more recent tool TACO^13^. This step is skipped if only one sample is provided. (4) Quantifies both newly predicted and known lincRNAs (defined in GENCODE) by featureCounts^14^, which generates read level (or fragment) digital expression. The combined known and lincRNA candidates can be analyzed by common tools such as edgeR^15^ or DESeq^16^. A normalized lincRNA expression by RPKM (reads per kilobase per million mapped reads)^17^ is also provided for quick and across gene comparison. (5) Annotates a lincRNA with a nearby protein coding gene along with the genomic distance between them. (6) Generates a summary html report with predicted lincRNA information and links to various reports.

The step 2 is the key for lincRNA discovery. Once transcripts are assembled, the pipeline compares the candidate transcripts with the provided known gene annotation (GENCODE by default) and selects those without any overlap with the known transcripts as novel transcript candidates. These candidates then go through several filters. The candidates that are shorter than 200 bp or single exon transcripts greater than 10000 bp are removed. For single exon candidates, those overlapping with repeat/low complexity regions of the genome (“simpleRepeat” downloaded from UCSC table browser at http://genome.ucsc.edu/cgi-bin/hgTables) are also filtered out. As transcript candidates at very low or high expression may be technical noises, the pipeline utilizes the statistical models of Sebnif to remove those transcripts not fully constructed (for multi-exon transcripts) or at extremely low or high expression (for single exon candidates) using the known multi-exon and single-exon transcripts as reference^7^. Noted is that the expression cut-off for single exon candidates is dynamically generated for each sample and is thus sample specific. Samples with low sequence depths would have lower absolute expression cut-offs than samples with higher sequence depths. The final step of the lincRNA prediction is protein coding potential evaluation. In addition to using Sebnif’s iSeeRNA module^8^, we provides CPAT, a popular tool developed by our group^12^ to allow users to choose the combination of the two for increased sensitivity (the union of non-coding transcripts from both) or specificity (intersection of the two). The reason we implemented these two tools was motivated by their observed complementarity. By default, we set non-coding potential greater than 0.9 for both programs; however, this can be reduced to as low as 0.5 for increased sensitivity as we learned from the known lincRNAs.

### Stranded RNA-seq

Strand specific RNA-seq protocol provides the coding strand information of each transcript. This protocol provides several advantages over the non-strand specific RNA-seq^18,19^ and is getting more common. It allows distinguishing the transcription activity of overlapping transcripts on the different strands so that all known lncRNAs (not just lincRNAs) can be more accurately quantified. It also can help to discover a new lncRNA candidate overlapping with other transcripts on the opposite strand. To utilize the features, the RNA-seq aligners have to have the capability to handle the strand specific data and a correct parameter is set in the alignment step. Both Tophat (2) and HISAT (2) can be used but HISAT2 is generally recommended as it is dramatically faster.

The pipeline goes through the similar steps as the non-stranded RNA-seq data except for the following: (1) the lncRNA candidate prediction would include the assembled transcripts that are overlapping with coding transcripts; (2) in the known lncRNA quantification, it includes all lncRNAs defined in the GENCODE annotation, which doubles the number of lincRNAs from non-stranded RNA-seq protocol.

### LncRNA annotation

LncRNAs are largely involved in the regulation of nearby or overlap protein coding genes (cis), although they can also regulate distant genes located on a different chromosome (trans). Hence, it is important to identify nearby genes with which lncRNAs may interact with, for downstream analyses. For this, we annotate all lncRNAs, both known and predicted, with nearby protein coding genes including their distance from its transcription start site (TSS).

### Pipeline implementation

The pipeline is developed in Linux environment using Java and shell scripts with third party modules (python, R, and C++). All needed modules and parameters are specified in configure files which allow users to customize their analysis. The workflow takes advantage of paralleled computing using Sun Grid Engine (SGE) for processing multiple samples simultaneously. The entire computing time and maximum memory usage are generally determined by the sample with the highest sequence depth and do not change too much to the number of input samples (but may be affected by the cluster load and performance). Thus, it normally takes a few hours to process hundreds of samples. Comprehensive log files are generated to monitor processes and report errors which help users to identify problems. It has been developed and fully tested with human reference genome hg19 but also works with hg38. The UClncR package and a test dataset can be downloaded from our website (http://bioinformaticstools.mayo.edu/research/UClncR). To avoid tedious installation for simple testing or to accommodate those without access to a cluster environment, a virtual machine version is also available where all dependencies are pre-installed.

### Test Data and pipeline evaluation

To evaluate the performance of UClncR, we used an ENCODE RNA-seq sample GM12878 (https://www.encodeproject.org/experiments/ENCSR000AEF), which was generated from stranded and PolyA enrichment protocol and sequenced at a high depth of 250 million pair-end 100 base reads. Sequence reads were aligned to the human reference genome (hg19) using HISAT2^20^ (v2.0.4, with following parameters–rna-strandness RF and –dta options). The aligned bam file was then processed by UClncR twice, one with intact GENCODE annotation (v19) to predict new potential lncRNAs not defined in the database and another with one third of lincRNAs removed to recover the known lincRNAs for performance evaluation.

### Complete UClncR function test using GM12878

This test was run with the complete GENCODE gene annotation (v19) and was used to evaluate the complete functionality of UClncR, as described above. When the complete annotation was provided to the pipeline, UClncR treated any transcript in it as known and predicted novel ones not defined in that annotation, which were either located in intergenic regions (lincRNAs) or in genic/intronic regions but on the opposite strand of known transcripts. The expression patterns of these predicted lncRNAs were compared with the known lncRNAs. The histone modification states of the coding regions of these lncRNAs were checked with the genome segmentation file generated by chromHMM algorithm^21^ for the exact same cell line, downloaded from UCSC (https://genome.ucsc.edu/cgi-bin/hgTables). The individual histone modification marks of H3K4me1, H3K27ac, H3K27me3 and H3K9me3 were also downloaded and compared with both known and predicted novel lncRNAs of this cell line.

### LincRNA candidate prediction by withholding part of known lincRNAs

Evaluating the accuracy of lncRNA prediction is very challenging since true lncRNAs are not known in a particular sample being predicted. To provide a solution, we applied a validation strategy where one third of known lincRNAs in GENCODE annotation (v19) were removed for UClncR to predict these lincRNAs using sample GM12878. To get a clean lincRNA set (note not all lincRNAs in GENCODE annotation pass filtering criteria) for the validation, we first ran all lincRNAs through the filtering of coding length, repeat region overlap, protein coding potential score and overlap with other transcripts and only kept those meeting the criteria, of which one third were withheld and remaining were passed to the pipeline along with all other genes. Among the withheld lincRNAs, we checked their coverage and only those with at least one read coverage were used as “truth set” to compare with the prediction result from the pipeline.

### TCGA lung adenocarcinoma

To illustrate the usability of the UClncR for a project with a large number of samples, we processed 583 TCGA LUAD (lung adenocarcinoma) RNA-seq samples of which 524 are from tumors and 59 from adjacent normal lung tissues. The downloaded fastq files were aligned using HISAT2 (2.0.4) with the non-stranded option as they were generated from non-stranded PolyA enrichment protocol. The aligned bam files were provided to UClncR for processing to identify both known and predicted lincRNAs.

## Results

**Figure 2:**
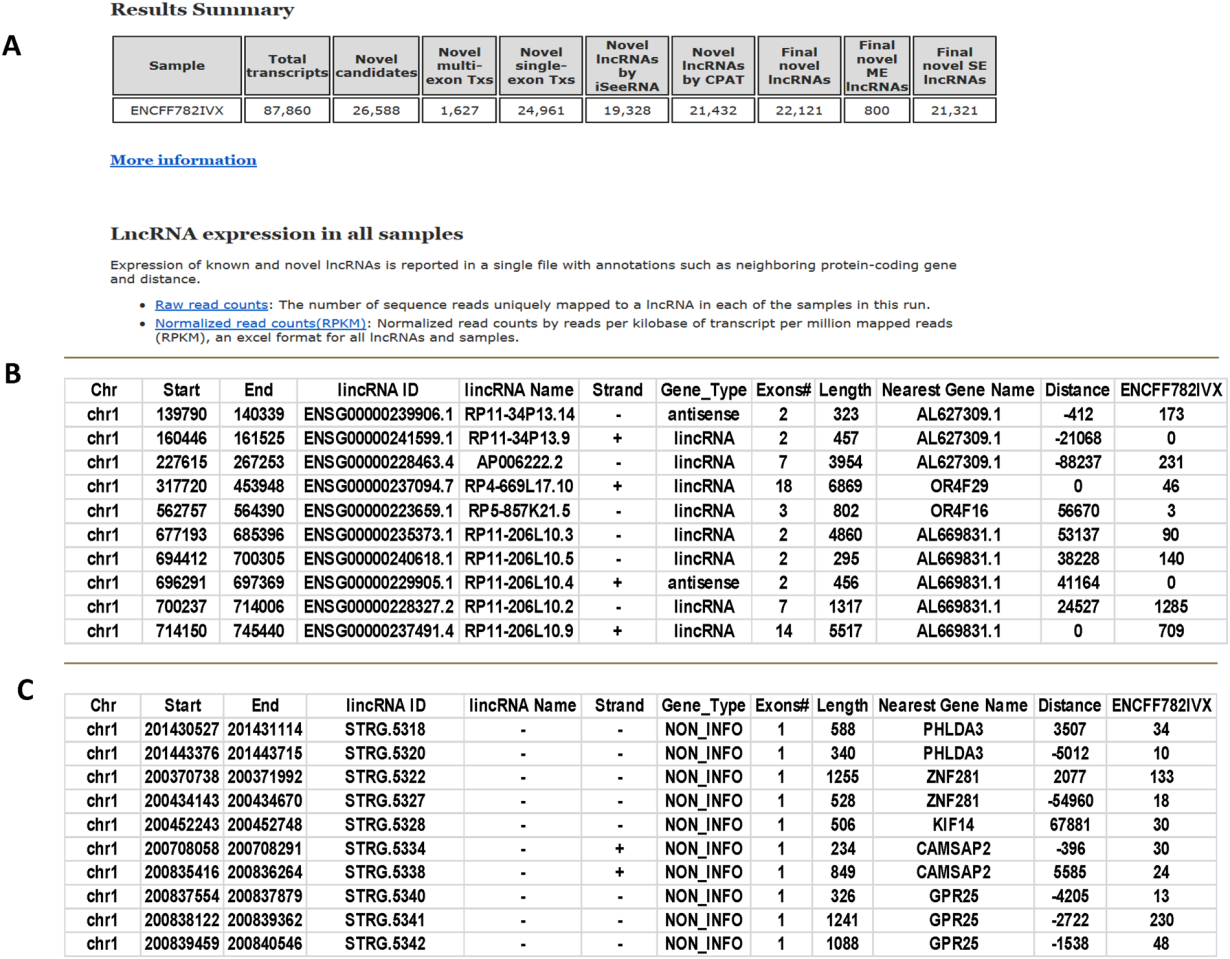
Pipeline output example. (**A**) Summary novel prediction for a sample. (**B**) Known lncRNA expression table. (**C**) Novel lncRNA expression table.

UClncRNA output (GM12878 as example). UClncR completed processing the high sequencing depth (~250 M reads) sample in less than an hour in our computing environment with 33GB max memory usage. The result is summarized in an html index page that starts with project description, configuration settings, and analytical workflow. The summary table provides information for number of transcripts assembled, number of lncRNA candidates after removing known transcripts, number of predicted single exon/multi-exon lncRNA transcripts after various steps of filtering and evaluations (Fig. 2A). The html page links to the main result of lncRNA expression matrix tables, which include both known and predicted lncRNAs at raw and normalized values (Fig. 2B,C). The table contains the transcript coding location, length, number of exons, nearby protein coding gene, and expression level for individual lncRNAs. Detailed information for each sample can be found under the “samples” directory. The final predicted lncRNAs with their transcript level expression, CPAT and iSeeRNA prediction scores can also be found in the “merge” subdirectory.

### Predicted lncRNAs in GM12878

**Figure 3:**
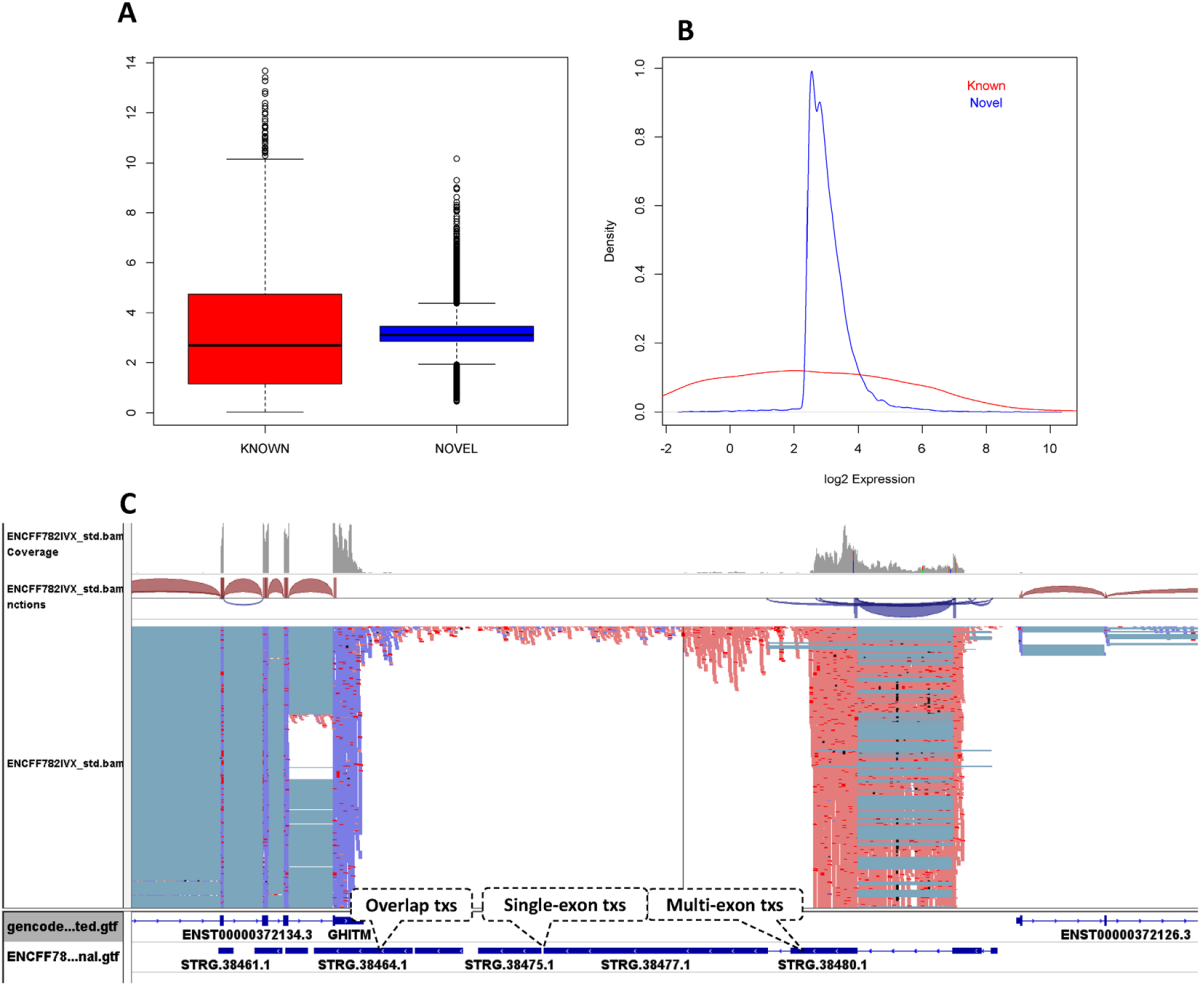
Novel lncRNA profile of the ENCODE RNA-seq sample. (**A**) Expression range (boxplot) of known and novel lncRNAs. (**B**) Expression density plot of known and novel lncRNAs. (**C**) Examples of predicted novel lncRNAs, including a multiple-exon one, a single exon one without overlapping and a simple exon one with overlapping in the opposite strand.

UClncR predicted 22,121 lncRNA transcript candidates from this ENCODE sample, 800 multi-exon and 21,321 single exon ones (Fig. 2A), which represent 21,794 unique lncRNA candidate genes. Only 5,568 out of 13,886 (40%) lncRNAs listed in GENCODE v19 had at least one read mapped (gene level expression > 0). Compared to the expression of known lncRNAs, the predicted ones had slightly higher median overall expression (3.1 vs. 2.38, Wilcoxon rank sum test p value < 2.2e-16; the mean of 3.24 vs. 3.16, t test p value 0.015) but narrower range (interquartile range) (Fig. 3A). Most of these lncRNAs were at low expression (log 2 values of 2~4, Fig. 3B). While the majority were single exon lncRNAs, 800 were multi-exon ones with exon-exon junction read support (i.e., a decent number of sequence reads mapped to the two exons which have been joined together, Fig. 3C). With the stranded RNA-seq, the pipeline was able to predict 5,459 lncRNA candidates that overlap with other coding genes (Fig. 3C), which would be missed in non-stranded RNA-seq data.

**Figure 4:**
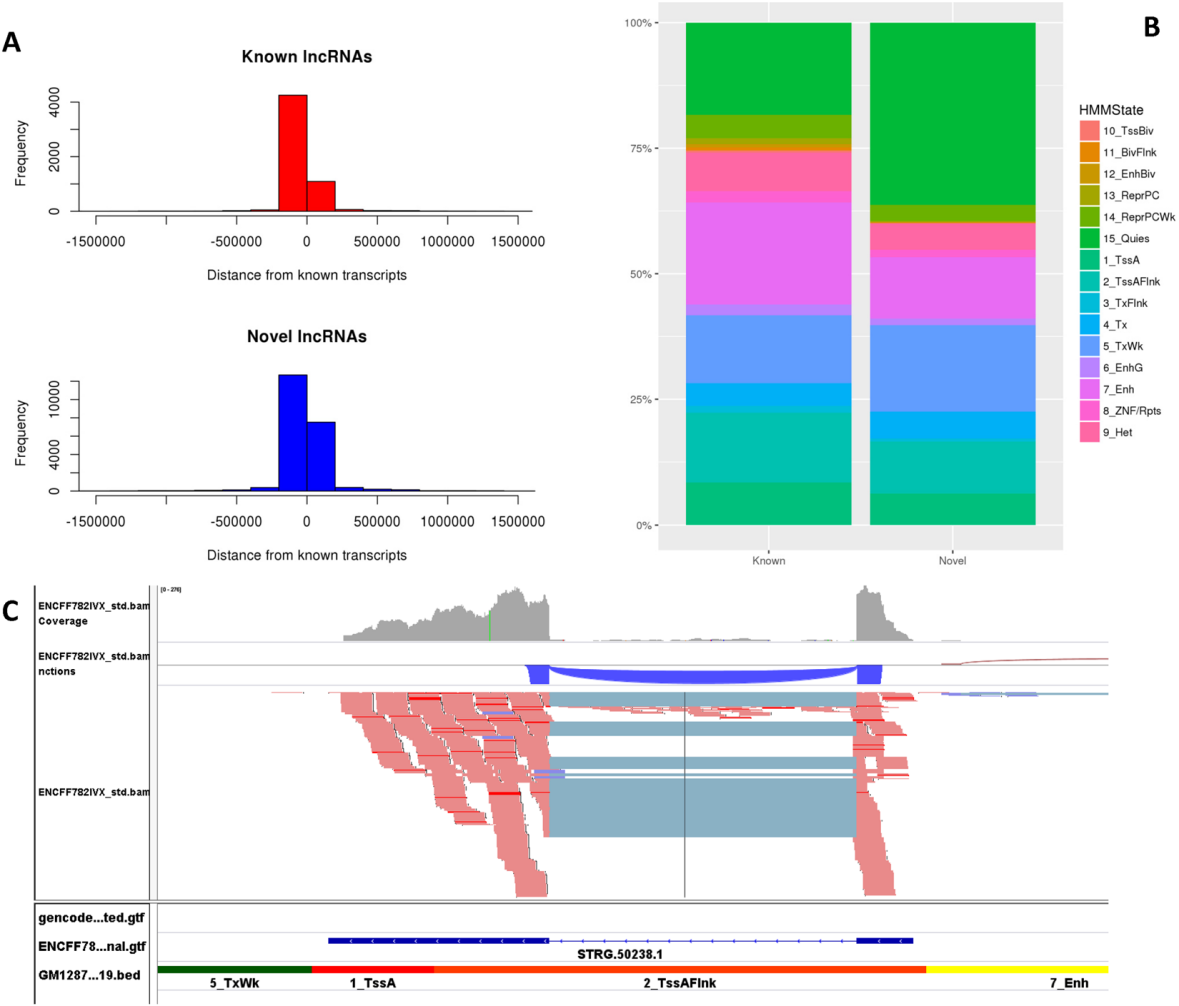
Novel lncRNA profile in relation to other genes and histone modification states. (**A**) Distances of novel and known lncRNAs to protein coding genes. (**B**) Overlap with chromHMM states. (**C**) An example of novel lncRNA with chromHMM states indicating its active transcription.

When the distances of the predicted lncRNAs to the closest protein coding genes were compared to those from known lncRNAs, we found similar distribution, i.e., the vast majority of them are within 250Kb of TSS of coding genes (Fig. 4A). We also compared the chromatin states of the predicted and known lncRNAs by overlapping chromatin segmentation states for the same sample and found that almost all the known or predicted lncRNAs (>99.9%) overlapped with one of the 15 chromatin states. About 55% of predicted lncRNAs were associated with active histone states (state 1–7) while about 65% were associated with known ones (Fig. 4B). For example a predicted lncRNA STRG.50238.1 is completely overlapped with Flanking Active TSS (2_TssAFlnk) region (Fig. 4C). Some of lncRNAs may be enhancer-derived RNAs (eRNAs), thus we also checked the overlap of these predicted multi-exon lncRNAs with each of the following histone modification marks: H3K4me1, H3K27ac (active marks, more in enhancers), H3K27me3, and H3K9me3 (repressive marks). Not surprisingly, we found that 90.3%, 82.4%, 32.6%, and 72.3% of these novel lncRNAs overlapped with above marks, respectively, while the overlap with the known lncRNAs was 86.4%,71.6%, 54.3%, and 65.8%.

### LincRNA prediction by withholding part of known lincRNAs

**Figure 5:**
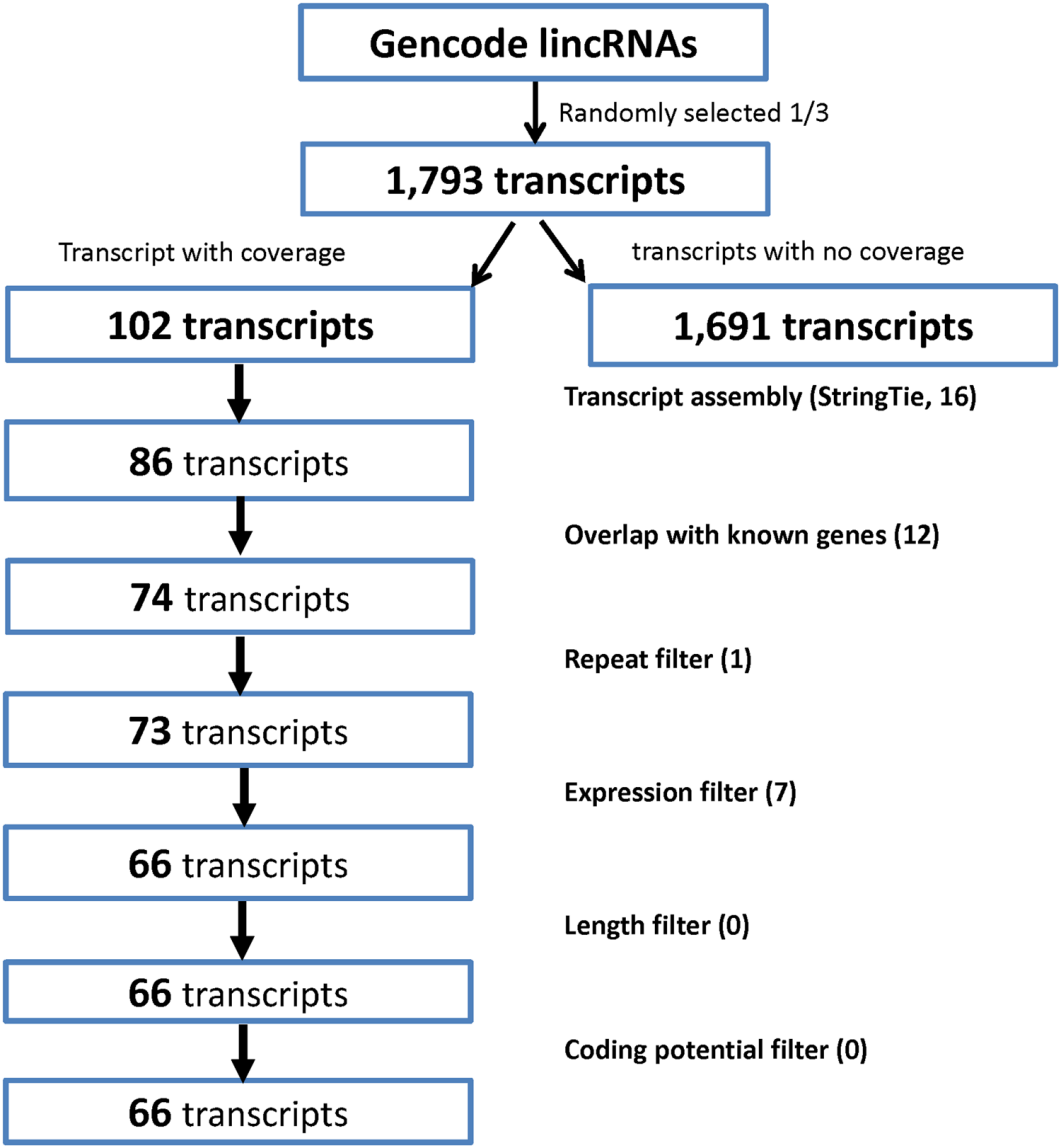
Prediction evaluation of the withheld known lincRNAs in sample GM12878. One third of known lincRNAs were withheld and their expression was evaluated. Only those with expression were kept for assessment. The number in each box is the number of transcripts remaining from the previous step and the text at right indicates the specific step in the pipeline with the number filtered out at that step within parenthesis.

Out of the 1793 lincRNA transcripts withheld for novel prediction, 102 had some read coverage in GM12878 RNA-seq data and therefore could be potentially predicted. Upon completion of the pipeline, 86 transcripts had sufficient expression and were successfully assembled by StringTie, of which UClncR reported 66 “novel” lincRNA transcripts and 12 lncRNAs overlapping with nearby genes (the recall rate of 90.7%). The missed transcripts included 1 that was filtered out due to overlap with a repeat region of the genome and 7 that did not pass the expression level filter of the pipeline (Fig. 5), which is a configurable parameter. Lowering the threshold would increase the sensitivity but reduce the specificity in real practice.

### TCGA Lung Adenocarcinoma

**Figure 6:**
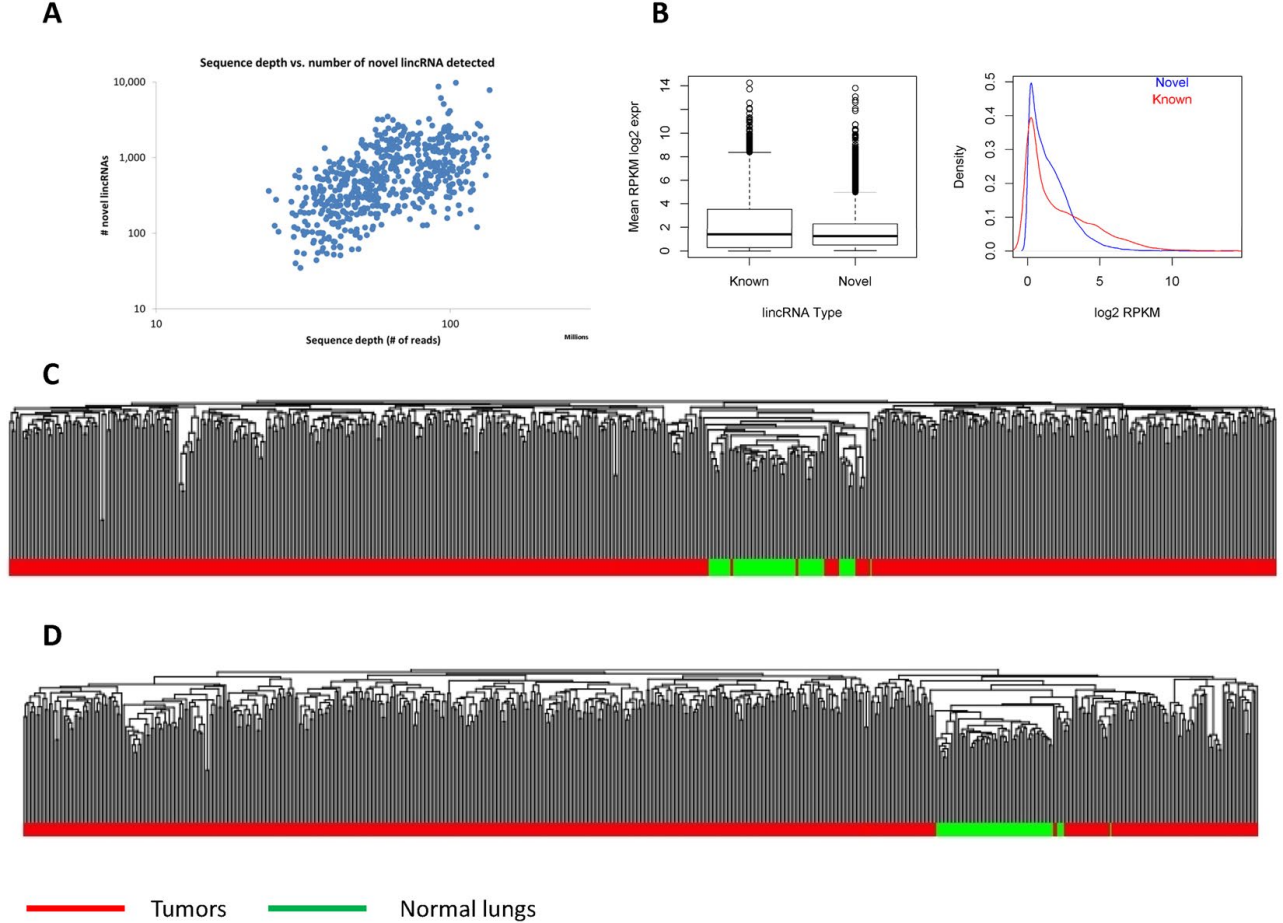
Novel lincRNAs in TCGA lung cancer (adenocarcinoma) samples. (**A**) Sequence depths (millions of reads) vs. number of novel lincRNAs predicted. (**B**) Expression profile of novel lincRNAs vs. known lincRNAs. (**C**) Unsupervised clustering for all samples using novel lincRNAs shows clear separation of tumors from normal tissues. (**D**) Unsupervised clustering for all samples using known genes shows the similar patterns as those from novel lincRNAs.

UClncR completed the processing and analysis of the 583 TCGA LUAD samples in less than 3 hours in our parallel computing environment. As the RNA-seq is non-stranded, only lincRNAs were quantified and predicted. The highly variable sequencing depths from sample to sample (ranging from 24 to 136 million pair end reads) led to varied novel predictions in each sample (ranging from 35 to 9,762), underlying the impact of sequencing depth on novel lincRNA discovery (Fig. 6A). The total number of novel lincRNA candidates from the 583 samples was 51,877, of which 5,014 were multi-exon ones.

**Figure 7:**
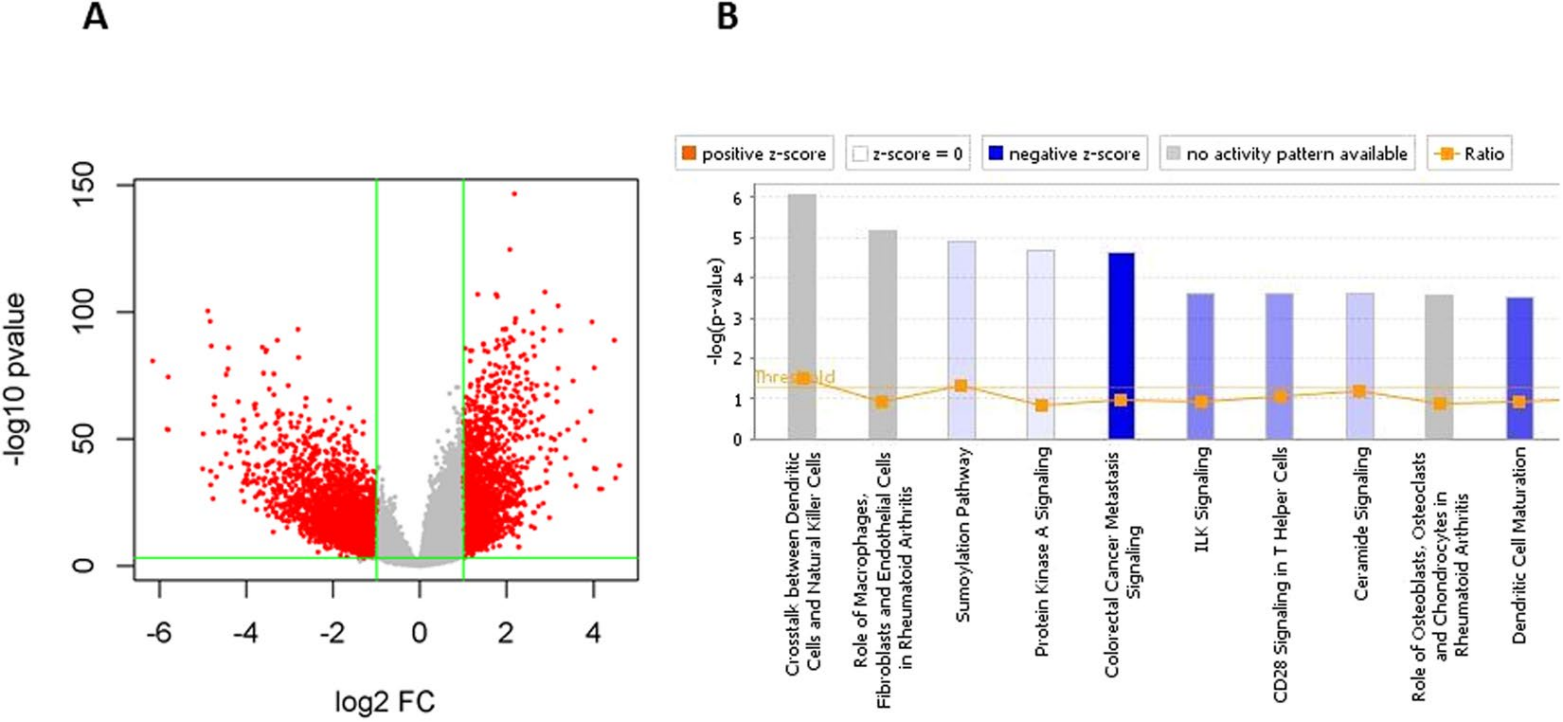
Differentially expressed novel lincRNAs in TCGA lung adenocarcinoma. (**A**) Volcano plot of differentially expressed novel lincRNAs. The red dots are those with p value less than 0.001 and log2 fold change >1. (**B**) Pathway analysis of the nearby protein coding genes for differentially expressed novel lincRNAs. The top pathways are involved in cancer development and immunity.

When compared with the known lincRNAs, the predicted lincRNAs had lower overall expression (Fig. 6B). Based on the expression of these lincRNAs, we conducted unsupervised clustering analysis for all the samples and found that tumor and normal samples formed distinct clusters (Fig. 6C). The same analysis was performed using all known genes and similar cluster patterns were observed (Fig. 6D), suggesting that similar to the annotated genes, these predicted lincRNAs had different expression patterns between tumors and normal samples. We further conducted correlation and co-expression analysis between lincRNAs and their associated protein coding genes. The correlations were mostly positive for both predicted and known lincRNA (Supplementary Figure S1). The lincRNAs and their corresponding genes also demonstrated similar expression patterns from the heatmap (Supplementary Figure S2). Majority of the predicted lincRNAs were differentially expressed between tumors and normal lung tissues (32,271 out of 51,877 at FDR < 0.05; 5487 with fold change > 2 and differential p value < 0.001, Fig. 7A). As a comparison, 32,400 out of 55,726 (Gencode v19, with expression in at least one sample) known genes were differentially expressed between tumors and normal lung tissues at FDR < 0.05 (58.2% for all genes and 52.7% for known lincRNAs), which is the similar proportion as the predicted lincRNAs. For the 5,487 differential novel lincRNAs with greater than 2 fold changes, 1,900 known protein coding genes were within 10KB of distance. Pathway analysis of these genes showed that they were highly enriched in cancer process and immune response (Fig. 7B).

## Discussion

Herein we describe UClncR, a comprehensive lncRNA discovery and analysis pipeline, which takes advantages of the fast transcript assembly tool, more complex lncRNA prediction algorithms, and parallel computing. Both predicted and known lncRNAs are reported for comprehensive lncRNA profiling. When RNA-seq is stranded, not only lincRNAs but also overlapping transcripts (i.e., all lncRNAs) are detected, which doubles the number of transcripts. For a project with many samples, UClncR can complete analysis in a matter of hours and generate a consolidated report for all samples so that further analysis will be dramatically facilitated using analytical tools routinely used for protein coding genes.

Although several studies report “novel” lncRNA discovery from RNA-seq with description of their methods^4,22–25^, none of them have an implemented software package in the public domain that can be easily accessible for comparison. Noted is that almost all use the older Cufflinks as transcript assembler, which is extremely slow (took nearly a week for a typical RNA-seq library) and the new StringTie provides significant performance gains in terms of speed and assembling accuracy^11,26^. In our testing for the NA12878 RNA-seq sample, StringTie completed assembly in 40 minutes while Cufflinks took 4 days and 5 times more usage of memory (Supplementary Table S1). For assembled transcripts/genes, Cufflinks generated three times more transcripts but fewer unique genes than StringTie (Supplementary Table S2), consistent with the previous evaluation that StringTie produces more complete and accurate reconstructions of transcripts/genes compared to Cufflinks^12,26^. With the integration of StringTie into the workflow, UClncR not only significantly reduces assembling time but also would increase prediction accuracy. However, evaluating lncRNA prediction accuracy from a real sample is really challenging as the truth is not known. In this manuscript, we selected a subset of known lincRNAs defined in Gencode as the “truth”; however, majority of them were not expressed in this particular sample and for those with some expression, it was difficult to define which ones had sufficient expression that could be assembled as a transcript by the tool like StringTie or Cufflinks. When these factors were considered, UClncR demonstrated the prediction accuracy of 90%. In real practice, many parameters can be adjusted for increased sensitivity or specificity in the pipeline.

Although Cuffmerge is used as default in our pipeline for meta transcript consolidation across samples, users can choose “merge” function implemented in StringTie or a more recent tool TACO^13^ as they may provide improved accuracy. However, from our initial testing, they appear to perform quite similarly. Among 2,947 predicted transcripts from 10 normal lung samples, 2831 (96%) were commonly assembled by all three tools (Supplementary Figure S3).

While UClncR provides a convenient way to discover and analyze both known and novel lncRNA candidates, novel lncRNA discovery is a challenging task and several important factors that potentially affect the discovery need to be considered. Most novel lncRNAs are expressed at a low level and high sequence depth is needed to discover them. Although there are no well-established requirements, our experience suggests a minimum of 100 million reads may be needed for reliable lncRNA prediction. Longer sequence reads (at least 100) is also recommended for more accurate alignment and transcript assembly. RNA-seq library preparation methods/protocols also have a significant impact. While PolyA selection method is clean and highly enriched for mRNA transcripts, lncRNAs that are not PolyA tailed do not get sequenced for discovery and quantification as a result. Total RNA with ribosome RNA removal method provides a better opportunity for novel discovery; however, even higher sequence depth is required as data from this protocol tends to contain more unusable reads (such as higher proportion of ribosome RNAs, un-spliced transcripts, and etc.). Stranded RNA-seq is more preferred to detect and quantify overlapping transcripts.

RNA-seq data quality may also have an impact on novel lncRNA discovery. The pipeline itself does not have capability to distinguish true transcripts from artifacts such as DNA contamination. Transcript assembly does not work well on degraded and fragmented RNAs as it is very difficult to assembly original and complete transcripts.

Lastly but importantly, one has to bear in mind that UClncR provides the first step of novel discovery for uncharacterized lncRNAs; however, they are only lncRNA candidates and they need to be further analyzed, filtered, and validated either by a different technology or in a new set of samples. The initial candidate list can be very long when a fair number of samples are predicted together, particularly for single exon ones. In our lung cancer dataset, over 50,000 candidates were predicted but only 5,000 were multi-exon lincRNAs. One of the common approaches is to limit those detected in multiple samples or focus on multi-exon lincRNAs only as single exon candidates can be artefactual background noise, un-spliced pre-mRNA or gene extensions^24,25^. UClncR just relieves the daunting work of initial discovery and allows investigators to spend more time on further analysis and interpretation of these findings.

## Conclusions

UClncR is a fast and convenient pipeline for users to detect and analyze long non-coding RNAs from RNA-seq, both known and predicted ones. It works with both stranded and un-stranded RNA-seq protocols and is particularly useful for a project with many samples so that they can be analyzed together swiftly.

## Electronic supplementary material

Supplementary Information
