# Author Correction: UClncR: Ultrafast and comprehensive long non-coding RNA detection from RNA-seq

**DOI:** 10.1038/s41598-018-23183-y

**Published:** 2018-03-20

**Authors:** Zhifu Sun, Asha Nair, Xianfeng Chen, Naresh Prodduturi, Junwen Wang, Jean-Pierre Kocher

**Affiliations:** 1 Division of Biomedical Statistics and Informatics, Department of Health Sciences Research, Mayo Clinic, Rochester, MN 55905 USA; 2 Department of Health Sciences Research & Center for Individualized Medicine, Mayo Clinic, Scottsdale, AZ 85259 USA; 3 Department of Biomedical Informatics, Arizona State University, Scottsdale, AZ 85259 USA

Correction to: *Scientific Reports* 10.1038/s41598-017-14595-3, published online 27 October 2017

The Acknowledgements section in this Article is incomplete.

“This work is supported by the Mayo Clinic Center for Individualized Medicine.”

should read:

“This work is supported by the Mayo Clinic Center for Individualized Medicine. The results published here are in whole or part based upon data generated by the TCGA Research Network: http://cancergenome.nih.gov/”

