# Supplementary material for: UClncR: Ultrafast and comprehensive long non-coding RNA detection from RNA-seq
Source: Sci Rep. 2017 Oct 27;7:14196. doi: 10.1038/s41598-017-14595-3 (PMC5660178; doi:10.1038/s41598-017-14595-3)
Supplement: Supplementary file 1 — Supplementary Information [file 41598_2017_14595_MOESM1_ESM.pdf]

UClncR: Ultrafast and comprehensive long non-coding RNA detection from RNA-seq

Zhifu Sun[1,#,*], Asha Nair[1,#], Xianfeng Chen[1,2,#], Naresh Prodduturi[1], Junwen Wang[2,3] and Jean-Pierre Kocher[1,*]

[1]Division of Biomedical Statistics and Informatics, Department of Health Sciences Research, Mayo Clinic, Rochester, MN 55905, USA; [2]Department of Health Sciences Research & Center for Individualized Medicine, Mayo Clinic, Scottsdale, AZ 85259, USA; [3]Department of Biomedical Informatics, Arizona State University, Scottsdale, AZ 85259, USA

#Contributed equally to the work; * Corresponding authors: sun.zhifu@mayo.edu and kocher.jeanpierre@mayo.edu

Supplementary Data

Figure S1: Correlation coefficient distribution between lincRNAs and their associated genes. The correlation coefficients are mostly positive (>0) for both novel and known lincRNAs and the correlations are higher in tumor tissues than in the normal tissues.

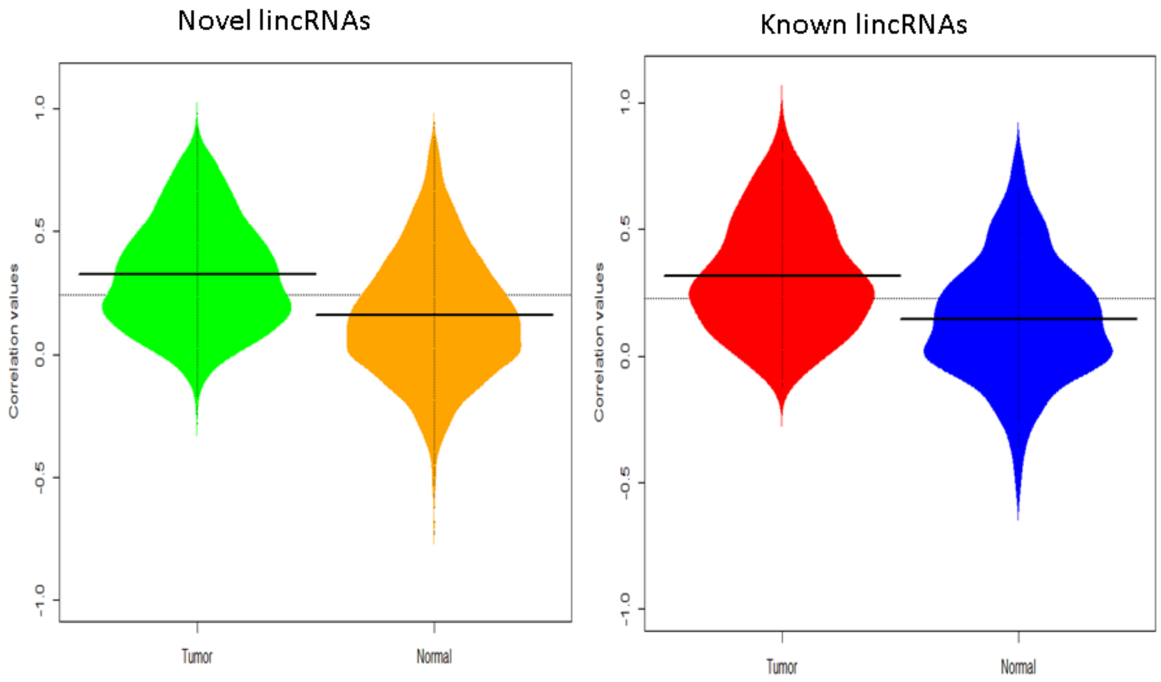

Figure S2: Co-expression heatmap between novel lincRNAs and their associated protein coding genes. For the differentially expressed lincRNAs between tumors and normal tissues, their expression is demonstrated by heatmap and the unsupervised clustering (left). For their corresponding protein coding genes, unsupervised clustering is done for samples only and the protein coding gene expression is kept in the sample order as corresponding lincRNAs (right). The co-expression patterns can be clearly seen between the two.

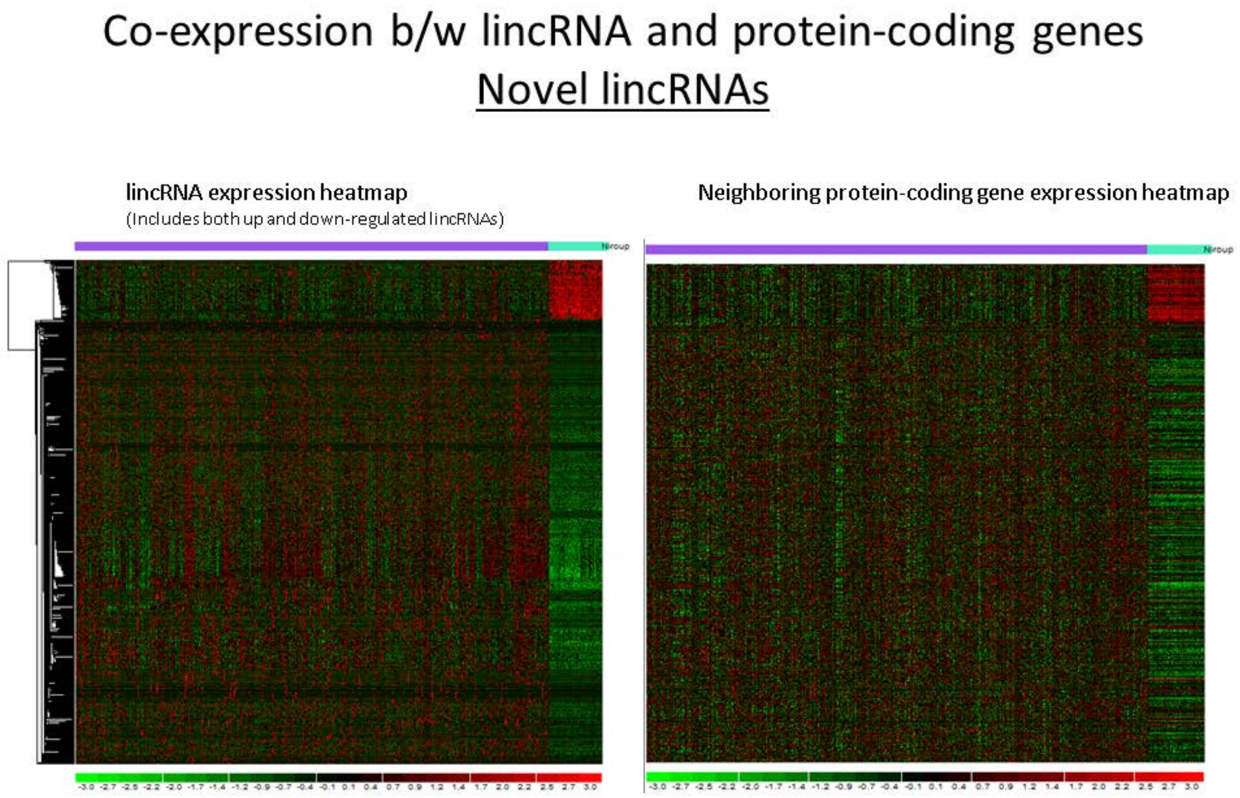

Figure S3: Transcript merging comparison from Cuffmerge, Stringtie and TACO. In this testing, we run 10 normal lung RNA-seq samples selected from TCGA dataset. Among 2,947 novel transcripts, 2831 (96%) are commonly assembled by all three tools. StringTie merge appears more accurate as it only 2 unique transcripts. However, the differences among the three are small.

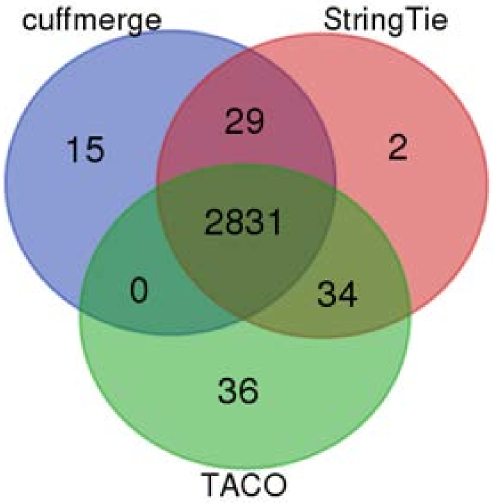

Table S1: Run time between Cufflinks and StringTie for NA12878

	Wallclock time	CPU time	No. of threads	Max virtual memory
StringTie	39 mins	48 mins	4	4.4G
Cufflinks	4 days	15 days	4	22.4G

Table S2: Transcript assembly comparison between Cufflincs and StringTie

	Cufflinks	StringTie
Total number of genes	55,927	69,120
Total number of transcripts	246,152	87,171
Average no. transcripts per gene	4.4	1.3
No. of multi-exon transcripts	176,678	32,384
No. of single-exon transcripts	69,474	54,787
